# Reversibility and Viscoelastic Properties of Micropillar Supported and Oriented Magnesium Bundled F-Actin

**DOI:** 10.1371/journal.pone.0136432

**Published:** 2015-08-31

**Authors:** Timo Maier, Tamás Haraszti

**Affiliations:** 1 Max Planck Institute for Intelligent Systems, Department of New Materials and Biosystems, Heisenberg str. 3, D-70569 Stuttgart, Germany; 2 University of Heidelberg, Institute of Physical Chemistry, Department of Biophysical Chemistry, Im Neuenheimer Feld 253, D-69120 Heidelberg, Germany; Karolinska Institutet, SWEDEN

## Abstract

Filamentous actin is one of the most important cytoskeletal elements. Not only is it responsible for the elastic properties of many cell types, but it also plays a vital role in cellular adhesion and motility. Understanding the bundling kinetics of actin filaments is important in the formation of various cytoskeletal structures, such as filopodia and stress fibers. Utilizing a unique pillar-structured microfluidic device, we investigated the time dependence of bundling kinetics of pillar supported free-standing actin filaments. Microparticles attached to the filaments allowed the measurement of thermal motion, and we found that bundling takes place at lower concentrations than previously found in 3-dimensional actin gels, i.e. actin filaments formed bundles in the presence of 5–12 *mM* of magnesium chloride in a time-dependent manner. The filaments also displayed long term stability for up to hours after removing the magnesium ions from the buffer, which suggests that there is an extensive hysteresis between cation induced crosslinking and decrosslinking.

## Introduction

Actin is one of the most abundant cytoskeletal proteins with crucial roles in maintaining the cellular shape, elasticity, adhesion and motility [1–3]. In its natural monomer form it is a globular protein (G-actin), but in cells and under proper buffer conditions these globular units dynamically assemble into filaments (F-actin). Numerous previous studies have analyzed the morphology and rheological properties of actin gels. In vitro, actin filaments can reach a length of ~20–30 *μm*. These studies show that the actin filaments possess a negative net charge with a linear charge density of about 4 *e* ^−^/*nm*, a helical structure with a twisting increment of ~36 *nm/turn* and a diameter of ~7–9 *nm* [2, 4–6]. Mechanically, actin filaments are semiflexible, with a persistence length in the order of 8–17 *μm*[7, 8], which is about 1/4–1/2 of their contour length, depending on the presence and type of the stabilization agent.

The rheological properties of actin gels depend on the concentration of actin and the crosslinker, as well as the chemical composition of the latter [9–16] (for a summary see Ref. [17]). While chemistry can be precisely controlled in these in vitro gel experiments, determination of network formation dynamics is greatly hindered because polymerization and bundling (crosslinking) occur simultaneously. Nevertheless, several physical and chemical parameters of network formation have been identified in such actin gels. For example, using light scattering of 0.5 *mg*/*ml* (c.a. 12 *μM*) actin solutions, Tang and Janmey showed that actin is bundled by divalent cations at various concentrations, i.e. *Co* ^2+^ at 5.5 *mM*, *Mn* ^2+^ at 7 *mM*, *Ca* ^2+^ at 20 *mM* and *Mg* ^2+^ at 27 *mM*[18]. Later, experiments indicated that the elasticity of isotropic and nematic actin gels depend on the magnesium concentration at much lower values than in the previous study, but a detailed correlation was not investigated [19]. Furthermore, experiments using actin comets, protrusion systems formed by polymerizing actin bundles, also indicated an effect at low magnesium concentrations [20].

The aforementioned studies have described the rheological properties of actin gels by modeling the filaments as linear, charged, semiflexible polyelectrolytes interacting with the divalent cations according to the counterion condensation theory. However, emerging information suggests a deviation from this classical mechanism, indicating the ability of divalent cations to promote bundling of filaments at lower concentrations than the previously estimated critical values. Therefore, anchoring actin filaments on supporting pillars or microparticles allows for dynamic control of the chemical environment resulting in time dependent control of the morphology and mechanical properties of the network [4, 21–23]. While this anchoring helps separate the polymerization and bundling processes, it also imposes a specific mechanical constraint: the diffusion of the filaments is hindered, and the bundling evolves in a characteristic zippering manner. Qualitatively, this behavior is independent of the bundling agent, thus various actin binding proteins (e.g. *α*-actinin, fascin, filamin, myosin-II) as well as divalent cations (*Ca* ^2+^, *Mg* ^2+^) exhibit a similar process [22].

Using microfluidics, one can estimate the binding energy from quantitative imaging, and subsequently conclude that the interaction energy of filaments is high, and the resulting Y-shape remains stable [4, 24]. However, the radius of the bending is not resolvable with optical microscopy, hindering a more exact estimation of the bending energy of the filaments in this state. A direct measurement of the forces driving bundle formation at the single filament level revealed very small values, on the order of 0.1–0.2 *pN*[25], with these measured forces exhibiting a saturation-like dependence with increasing cation concentration (for these experiments, the applicable magnesium concentration was limited to about ~25–200 *mM*). Thus, on one hand, experiments have shown filaments bundle into a stiff structure, which must be held together by large enough forces to resist unbundling [24]. On the other hand, the forces acting between individual filaments during bundle formation are on the order of sub-piconewtons [25]. This apparent contradiction can be resolved by assuming that the bundling process is driven both by weak forces and the semiflexible nature of the actin filaments; therefore, local changes of the molecules (e.g. conformation changes) increase the bundle stability, in a similar manner as magnesium or calcium do to the structure of G-actin [26]. Based on this assumption, we can hypothesize that actin bundles formed by magnesium ions would exhibit long term stability, and that the speed of bundling would depend on the concentration of the available divalent cations at low concentration values. In this paper, we present the time dependence of actin bundling due to low (2–12 *mM*), but constant concentration. We also present experimental evidence that this bundling cannot be reversed by removing magnesium from the buffer or by applying EDTA as a chelating agent to the bundled network.

## Materials and Methods

### Animal ethics statement

Rabbit muscle tissue was obtained from commercially available frozen rabbit meat, designated for human consumption. Thus, no live animals were utilized in this study, nor did the authors have contact with the meat producer. General commercial animal guidelines of Germany were met by the meat producer.

### Fabrication of microfluidic devices

**Fig 1:**
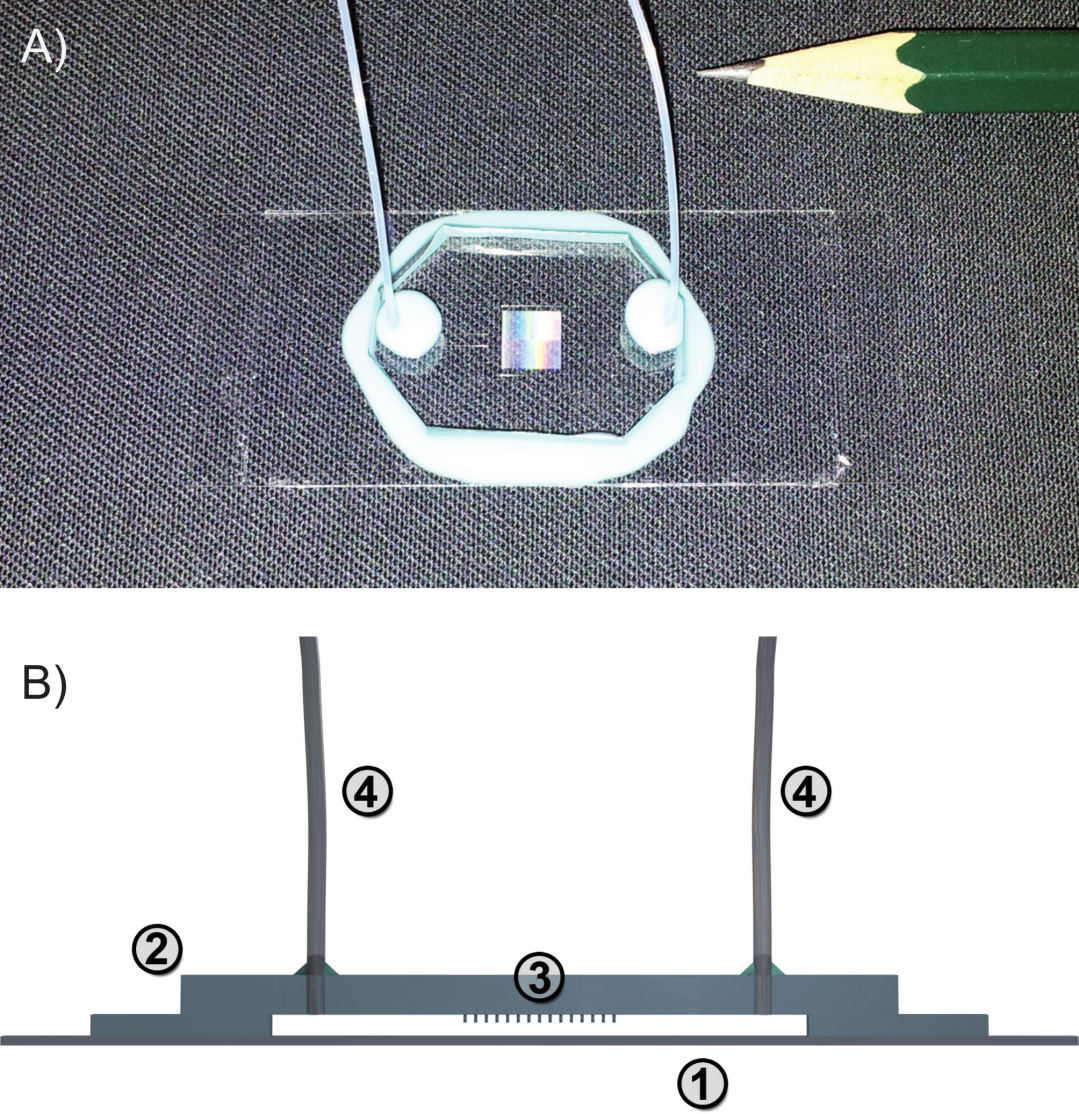
Flow channel for the creation of stress-fiber like actin structures. (A) A photograph of the prepared channel. (B) Schematic side view: The channel is imprinted into a thin PDMS layer on top of a cover slip (1) and closed with a ~3–4 *mm* thick block (2) with the pillar field (3) placed in the middle. The attached tubes (4) allow a continuous, pump driven exchange of liquid. (The scales are altered for better visibility.)

The flow channels (Fig 1) were constructed from two main parts: a main channel and a micropillar array protruding into the channel. The channels were typically 40 *μm* high, with a 6 *mm* × 6 *mm* main chamber in the middle. The pillars were 15 *μm* high with a diameter of 5 *μm* and a typical center-to-center distance of 13 *μm*. The pillar field was 5 *mm* × 5 *mm* in size, placed in the middle of the main chamber during assembly. Both parts were produced by standard soft lithography methods [27, 28] using SU-8 negative resists (MicroChem Corp., USA), treated according to the manufacturer’s instructions. Structures were exposed using a MJB3 mask aligner (SUESS MicroTec AG, Germany) equipped with a 350 *W* mercury lamp.

For the channel, a 40 *μm* thick layer of SU8-25 was applied onto a borosilicate glass slide (Krankenhaus- und Laborbedarf Manfred Fremdling, Germany), cleaned in aqueous Extran solution (Merck KGaA, Germany) (*Extran* : *H*_2_ *O* = 1 : 10 by volume) prior to coating. The pillars were made using a 15 *μm* thick layer of SU8-2010 on a silicon wafer (SI-MAT—Silicon Materials e.K., Germany) and a hole array structure, i.e. a chrome mask consisting of circular spots of 5 *μm* diameter and 13 *μm* center-to-center distance. After developing the masks with SU-8 developer (mrDEV-600 MicroChem Corp., USA), they were passivated by vapor deposition of 1H,1H,2H,2H-perfluorooctyltrichlorosilane (ABCR Dr. Braunagel GmbH & Co. KG, Germany) for 3 hours in a desiccator.

The mold was created by pouring polydimethylsiloxane (PDMS) (Sylgard 184, Dow Corning Corporation, USA) (*base* : *catalyst* = 10 : 1 by mass) onto the masks and, after subsequent degassing, hardened at 65 °*C* for 4 hours. For the pillar fields, a ~3–4 *mm* thick PDMS layer was created, while for the channels, a cover slip was pressed onto the mask, leaving a thin layer of PDMS around the structure of only a few microns. After peeling off the mold, a pair of PTFE tubes (Bohlender GmbH, Germany) were attached to the back of the PDMS pillar-block through holes made with biopsy punchers (Harris Uni-Core 0.5 *mm*, Ted Pella Inc., USA). Both the channel-coverslip as well as the pillar-block were then activated in oxygen plasma at a pressure of 0.5 *mbar* and 150 *W* for 30 seconds (100-E microwave plasma system, PVA TePla AG, Germany) to enable covalent binding between the two surfaces when assembled together. To ensure water tight seal, which is especially important at the PDMS-tube interface, the chamber was also sealed using “Twinsil 22 dublier-silicone” (Picodent Dental-Produktions- und Vertriebs-GmbH, Germany).

### Chemicals for buffers

If not stated otherwise, all buffer salts used for buffer solutions were purchased from Sigma-Aldrich Chemie GmbH (Germany) and used without further purification.

### Actin purification and polymerization

Actin was purified from rabbit skeletal muscle as previously described [29, 30] and mixed with biotinylated G-actin (Tebu-bio GmbH, Germany) in a 25 : 1 ratio leading to a final concentration of ~1.63 *mg*/*ml* in G-buffer (2 *mM* tris(hydroxymethyl)-ammomethan (TRIS), 0.2 *mM* *CaCl*_2_, 0.2 *mM* dithiothreitol (DTT), 0.77 *NaN*_3_, 0.2 *mM* adenosine 5’-triphosphate disodium salt (Na-ATP), pH 8.0). The samples were then flash frozen in liquid nitrogen and stored at –80°*C* until use. The actin concentration was determined using UV/VIS spectroscopy with an absorption coefficient of 0.63 *ml*/(*mg* *cm*) [31].

To achieve a 5 *μM* dilution of F-actin, polymerization was initiated by adding 12.5 *μl* G-actin to 10 *μl* of 10× F-buffer (20.0 *mM* TRIS, 20.0 *mM* *MgCl*_2_, 1000 *mM* KCl, 2.0 *mM* *CaCl*_2_, 2.0 *mM* DTT, 5.0 *mM* Mg-ATP; *pH* 7.4) and 72.5 *μl* *H*_2_ *O*. After 10 minutes, the filaments were stabilized and fluorescently labeled by adding 5 *μl* of a 0.1 *μM* tetramethylrhodamin-B-isocyanate labeled phalloidin (phalloidin-TRITC) solution.

### NEM-HMM

Heavy meromyosin (HMM) was prepared from rabbit muscle using the Margossian and Lowey method [32], with its ATPase functionality blocked using N-ethylmaleimide (NEM) according to a previously published protocol [33, 34], resulting in NEM-HMM which was used to bind actin filaments to the PDMS pillars.

### Preparation of actin networks

**Fig 2:**
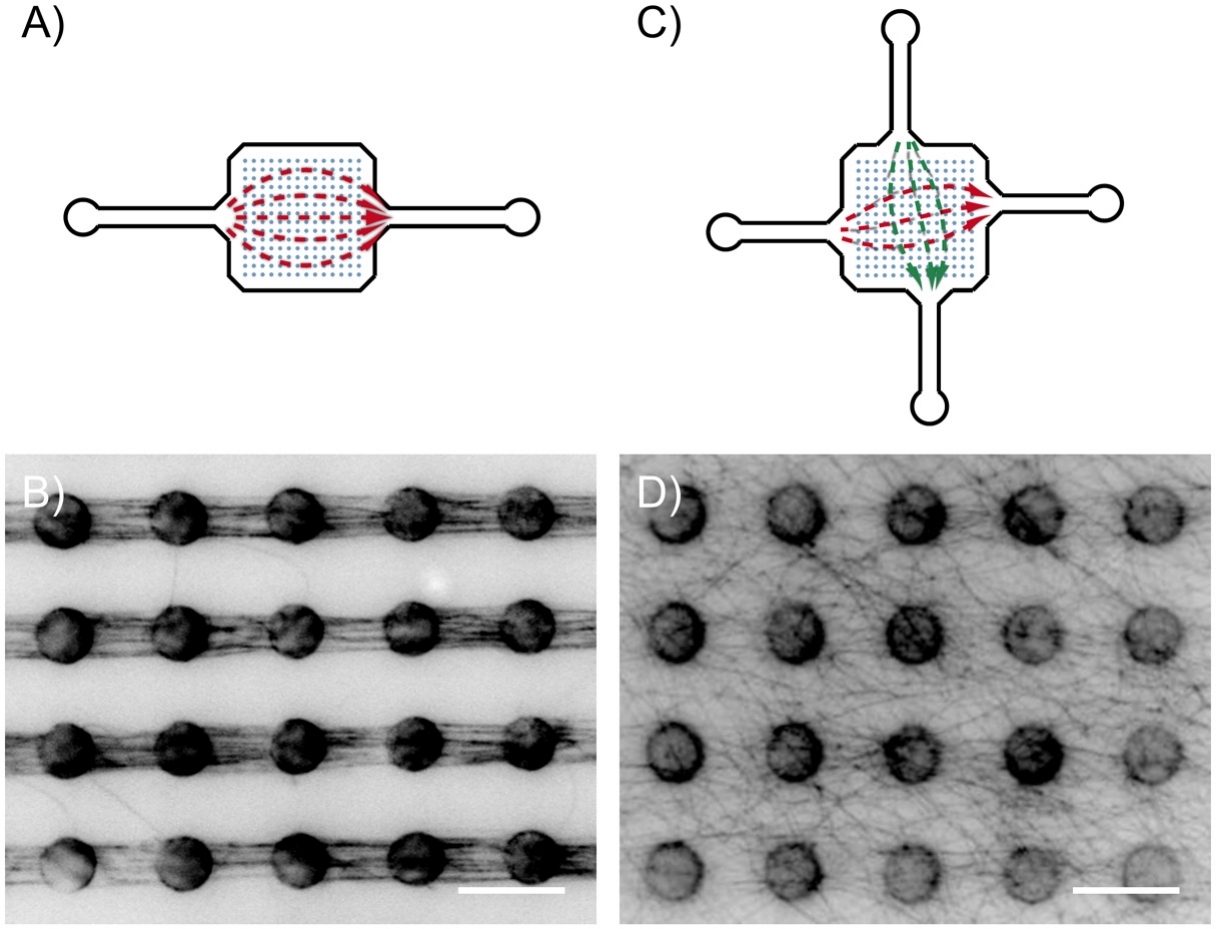
Flow channel designs and application. (A) A two-armed flow channel to form parallel bundles. (B) Actin structures formed from prepolymerized filaments. (C) A four-armed channel, where flow directions can be alternated (examples are indicated by the red and green arrows). Using alternating flow directions, very complex, randomly oriented networks can be achieved. (D) An example of a highly random network polymerized in-situ in the channel depicted in (C). (Scale bar is 10 *μm*.)

The flow channel was first rinsed with 30% aqueous ethanol solution and then with test buffer (T-buffer: 25 *mM* imidazole, 1 *mM* ethylene glycol bis(beta-aminoethyl ether)-N,N,N’,N’-tetraacetic acid (EGTA), 2 *mM* *MgCl*_2_, 25 *mM* KCl; *pH* 7.4) at 5 *μl*/*min* for 15 *minutes* each. Next NEM-HMM solution (5 *μM* in T-buffer) was flushed in to ensure the attachment of actin filaments to the pillar tops. Previous studies have shown that the NEM-HMM physisorbs to the PDMS pillars and remains throughout subsequent rinsing processes. After rinsing with T-buffer, a diluted F-actin solution (0.05 *μM*) was injected at a flow rate of 0.4 *μl*/*min* with a syringe pump (Pico Plus, Harvard Apparatus, USA). After reaching the desired filament density, the flow chamber was rinsed again with T-buffer to extract non-attached filaments (Fig 2).

In-situ polymerized networks were formed by injecting the polymerization solution into a 4-armed channel immediately after adding G-actin to the solution, allowing the polymerization to take place in the flow chamber. Excessive filaments were then extracted after an incubation time of 2 *hours* by rinsing the channel and alternating the flow direction, i.e. gradually lowering the frequency in 5 cycle steps from 0.05 *Hz* to 0.003 *Hz*. This procedure led to a much higher isotropy of actin orientation the filament adhesion method (see Fig 2).

### Buffers for actin bundling and ion removal

For cross-linking actin, T-buffer containing *MgCl*_2_ at concentrations of 2–12 *mM* was used, as well as a buffer for removing *Mg* ^2+^ ions (25 *mM* imidazole, 1 *mM* EGTA, 5 *mM* ethylenediaminetetraacetic acid (EDTA), 25 *mMKCl*; *pH* 7.4). The buffers were injected at a loading rate of ~0.4 *μl*/*min*. with an input tube length of 195±3 *mm*. Particle tracking experiments were started ~30 minutes after the start of injection, because the solution required ~34 minutes to pass through the tube.

### Microparticles

To characterize the thermal motion of the filaments, streptavidin coated polystyrene microbeads (PC-S-2.0, Kisker Biotech GmbH & Co. KG, Germany, stock concentration c.a. 0.2 *mg*/*ml*) with a reported diameter of ~1.87 *μm* were injected into the system at a 1 : 20 dillution in T-buffer.

### Microscopic imaging and analysis

The experiments were performed on a custom made microscope platform, built around an IX71 inverted microscope body (Olympus Deutschland GmbH, Germany), equipped with a custom illumination and imaging system. Sample positioning is controlled by a motorized stage (SCAN IM 112x74, Maerzhaeuser Wetzlar GmbH & Co. KG, Germany) and fine control is provided by a 3-axis piezoelectric stage (P-563.3CD, Physik Instrumente (PI) GmbH & Co. KG, Germany; working range 300 *μm* in the x,y,z-directions) built on top of the motorized stage.

The microscope was equipped with a Zeiss plan-NEOFLUAR water immersion objective (Carl Zeiss AG, Oberkochen, DE, 63×, numerical aperture *NA* = 1.2). Bright field imaging was done in transmission mode via a 660 *nm* collimated LED (M660L3-C1, Thorlabs, USA) light source, and an A602f CMOS camera (Basler AG, Germany) allowing for image acquisition up to a rate of 100 *fps*. Fluorescence imaging was carried out using a diode pumped solid state laser (DPSSL-70, Roithner Lasertechnik GmbH, Austria) emitting light at a wavelength of 532 *nm*. The emission filter has a center wavelength of 570 *nm* (AHF Analysentechnik AG, Germany) and image recording was done using a Hamamatsu ORCA-ER CCD camera (Hamamatsu Photonics Deutschland GmbH, Germany). The camera and the laser were synchronized by a custom-built programmable trigger (based on an Olymex SAM7S-P64 board, Olymex Ltd, Bulgaria), providing TTL pulses with sub-millisecond accuracy. To minimize external oscillations the setup was placed on an actively damped optical table (VH3660W-OPT, Newport Co., USA).

All image acquisition was performed using the free dc1394 DCAM library for firewire cameras, and its graphical user interface program Coriander on a Linux workstation. Sample positioning was driven by self developed programs using the object oriented programming language Python. Particle tracking was done according to the principles and algorithm described by Crocker et al. using MATLAB [35, 36]. Computation of the viscoelastic moduli was accomplished by microrheology algorithms developed in our laboratory (see Ref [37]). The restoring potential and force constants were calculated using Boltzmann position probability in a harmonic potential well, fitted by a nonlinear least squares algorithm (leastsq from the Scientific Python library [38]).

Confocal microscopy was performed using a motorized Axiovert 200 microscope (Carl Zeiss AG, Germany) and the Pascal 5 LSM module. The imaging objective was the same as for the epifluorescence imaging.

### Time dependent bundling experiments

Once the system reached a steady state at the beginning and end of the experiments, trajectories were recorded for 5 *minutes* at 100 *fps* (30,000 images) using the transmission illumination. In-between, shorter datasets containing 5,000 positions were collected for 50 seconds, which was the minimum time considered to be necessary to obtain reasonable statistics. Fluorescence images were recorded separately before and after the measurements to check for the presence of an actin network.

## Results and Discussion

**Fig 3:**
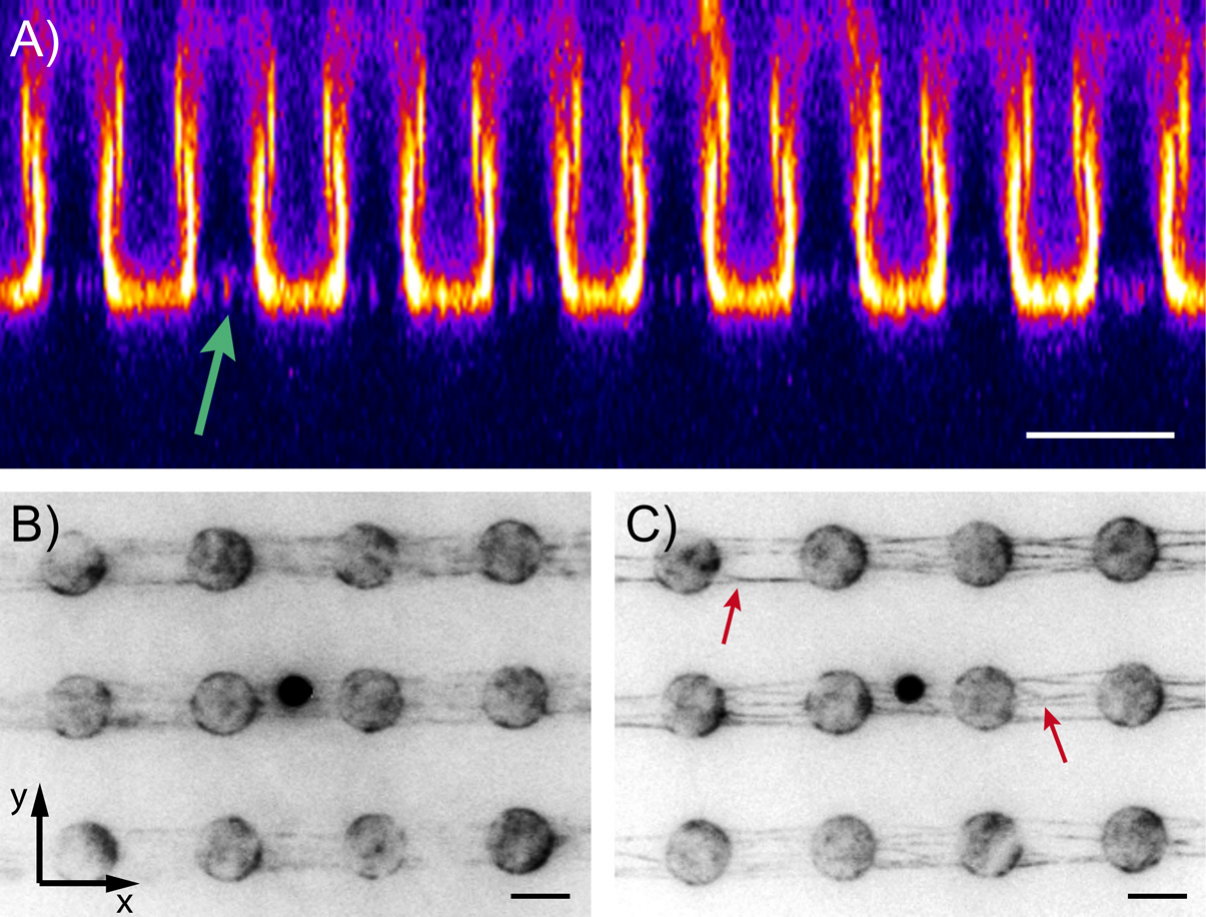
Actin networks produced on pillars. (A) A cross-section fluorescence image of an in-situ polymerized actin network located on top of PDMS micropillars. The pillars, which fluoresce due to adosrption of actin/phalloidin onto their surface, point from the ceiling towards the bottom of the flow cell. However, actin filaments are only located in a thin layer on the pillar tops. (B) While there are no single filaments visible in the non-cross-linked case due to thermal motion, (C) cross-linking with 50 *mMMgCl*_2_ shows the typical Y-shaped cross-linking structures (indicated by red arrows). (Scale bar is 5 *μm*.)

The micropillar flow channels presented here allow for the construction of quasi 2-dimensional actin networks on the pillar structures in a controllable manner. The morphology of the resulting actin network depends on the flow conditions and the type of filaments used, i.e. preformed or formed in the chamber (Fig 2). In all cases, we found that the network forms in close vicinity to the pillar tops (Fig 3A). This localization is likely due to the fact that the vertical drag, which can be characterized using the z-component (parallel to the axis of the pillars) of the average flow velocity, is minimal at the pillar tops, as it has been reported from particle velocimetry measurements in such channels [39]. These experiments have also proven that under constant flow, the medium is completely exchanged in the channel. In order to characterize the bundling properties, network structures containing parallel-oriented filaments were chosen, which were constructed using pre-polymerized actin. Due to the nature of this construction method, the actual orientation (i.e. the direction of the + or − ends of each filament) could not be controlled, therefore the parallel geometry contains a random set of actin filaments oriented in both parallel and ant-iparallel directions.

The mechanical properties of the bundles were determined by tracking the thermal motion of streptavidin coated polystyrene tracer particles (diameter about *d* ≈ 1.9 *μm*) attached to the biotin-labeled filaments (Fig 3). The strong streptavidin-biotin coupling ensures a permanent correlation between the motion of the particle and the bundle fluctuation. Because the coupling causes a local deformation of the bundle structure, the apparent stiffness of the unbundled filaments is expected to increase. The maximum value of this local deformation is ~30% of the length of a filament (estimated by comparing the particle size to the length of a free filament segment of about 8 *μm*). Particles chosen for tracking were bound to filaments that were approximately parallel to the x-axis of our viewing field, as well as located at the center of the gap between two PDMS micropillars. This latter criterion was used in order to minimize the effect of colloidal interaction between the tracer particles and the supporting posts. The elasticity of the attached bundle was determined by tracking the thermal motion of the tracer from image stacks recorded at various time points. The injection of the bundling medium was stopped for the duration of the recording.

### Anisotropic viscoelastic behavior

Bundling of the filaments using the test buffer, which contains a relatively high concentration (50 *mM*) of magnesium, results in a fast change to the system, inducing the well known zippering process [22, 25, 40]. The process ends with the characteristic splay filament configuration at single filament level (Fig 3C, red arrows), as well as bundle formation. The fluorescence images also indicate that this is an overall process, resulting from the change of buffer. The position distribution of the tracers was calculated from image stacks recorded before and after the injection of *Mg* ^2+^ ions. The data was then rotated using the second-momentum (or structure tensor) of the distribution, such that the x-axis is oriented along the filaments and y-axis perpendicular [37, 41]. Thus, in the x-direction, the bead moves or rolls along the filaments, and in the y-direction, the filaments bend, moving the bead with them. In this coordinate system, one can expect that the bundling process affects mainly the perpendicular, or y-component. As the filaments bundle, the structure attached to the particle becomes stiffer, resulting in a decrease of thermal motion in this direction.

**Fig 4:**
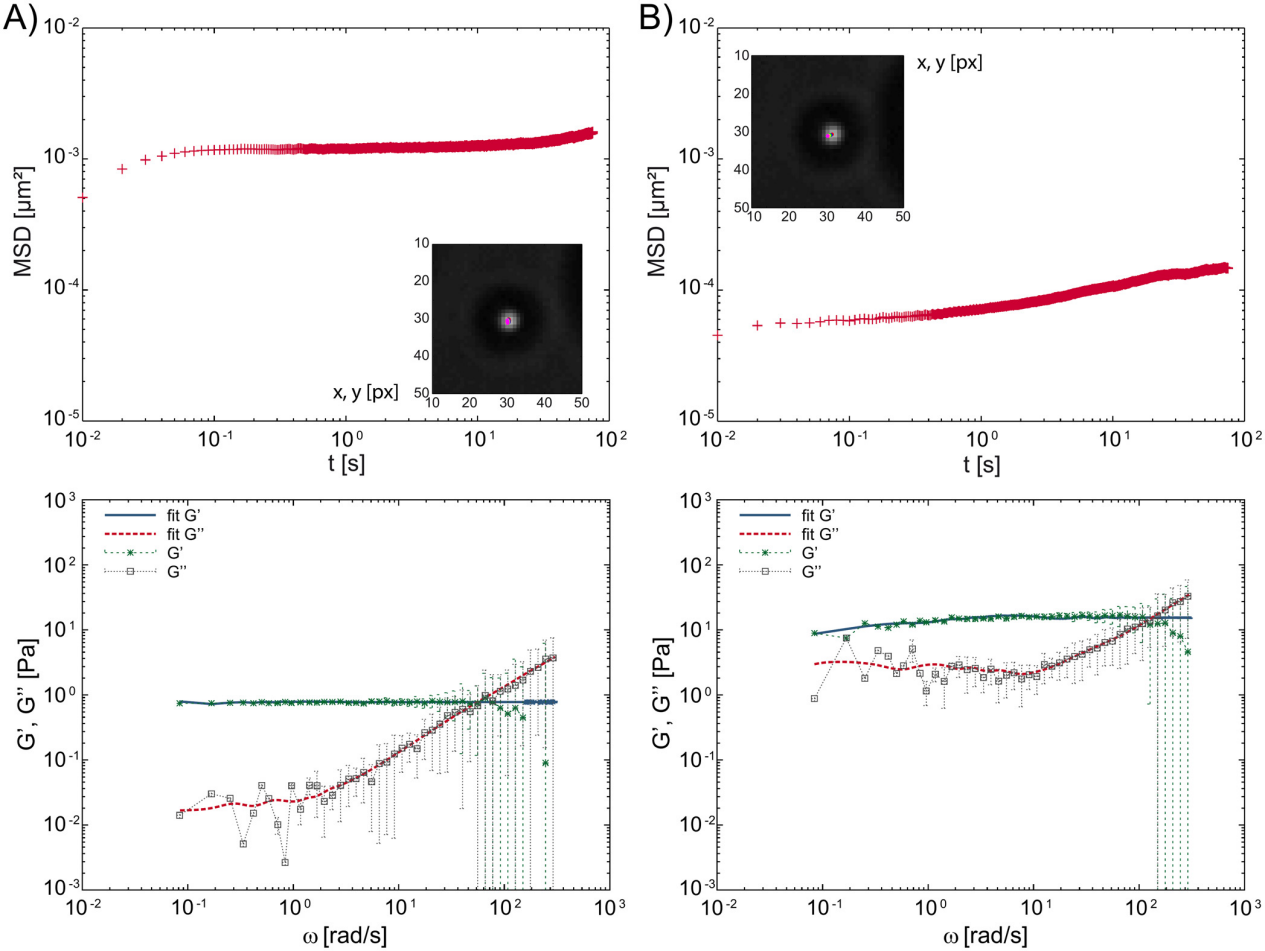
Particle tracking rheology analysis of actin bundles. MSD curves and frequency dependent viscoelastic moduli *G*′(*ω*), *G*″(*ω*) of a ~1.9 *μm* particle bound to anchored actin bundles on PDMS pillars. (A) Before (B) and after crosslinking using 50 *mMMgCl*_2_. The complex shear modulus is log-bin averaged, the errorbars showing the standard deviation.

Calculating the 2-dimensional mean squared displacement (MSD) using overlapping time intervals [37], the data can be split into two parts: the initial time points which can be fit using a model of the Kelvin-Voight elastic solid theory [42], and the later time points which are characterized by an exponential curve that plateaus and has an exponent on the order of a *α* ≈ 0.05 (Fig 4). Conversion to the frequency dependent complex shear modulus G*(*ω*) was done using both the direct conversion and the spline-like interpolation methods. The real part of the complex shear modulus is the storage modulus *G*′(*ω*), describing the elastic characteristics of the sample, and the imaginary part is the loss modulus, *G*″(*ω*), characterizing the viscous loss in the material [8, 10, 14, 15].

The storage modulus, *G*′(*ω*), exhibits a broad plateau, while the loss modulus *G*″(*ω*) shows a transition to a plateau at lower frequencies, similar to the characteristic minimum observed for entangled polymers [43, 44]. This nearly purely elastic response expected because F-actin is semiflexible, the experimental conditions limit the maximum filament length to less than the persistence length and the binding of filaments to the pillars eliminates the possibility of axial diffusion. Thus, the measured elastic response of these networks are due to a combination of the filament properties and the experimental construction.

Comparing the elastic moduli before and after bundling, we observed an increase of ~2–10 times during bundling (Fig 4), in agreement to previous observations on 3-dimensional actin gels [8, 14]. This trend was also present in the x- and y-components of the data (not shown). The change was smaller for the x-component, but it strongly depends on the amount of actin bound to the beads, which cannot be controlled in this experimental setup. While the bundling process can be clearly detected in the collected data, the quantitative evaluation exhibits a large standard deviation. This large error is due to the fact that while magnesium ions bind the filaments together, resulting in an increased elasticity, they also increase the probability of filaments directly binding to the beads, thereby causing a further increase in the response. The extent of this latter contribution is limited by the accessible amount of filaments and bead area, but cannot be quantified by current microscopy methods.

### Reversibility of the bundling

Assuming the condensation theory of divalent cations driving bundling, one would expect that the divalent ions bound to actin maintain a dynamic equilibrium of the bulk solution, and that the surface concentration follows changes of the bulk concentration. However, if the filaments form a strong bond, for example due to conformational changes, one would expect that no such equilibrium could exist; therefore removing ions from the buffer would be unable to recover the initial state.

In order to test this stability of the chelating agents (to remove divalent cations) were applied after filament bundling at low concentrations of *MgCl*_2_, i.e. 0–12 *mM*. Specifically, at least 1 *hour* after magnesium injection and after the bundling was completed, the bundling buffer was replaced with a magnesium-free solution containing 1 *mM* EGTA and 5 *mM* EDTA. This treatment was expected to remove any weakly bound magnesium or calcium ions present, but should not affect ions which are already strongly bound between the filaments. 3 *hours* after the treatment, particle positions were recorded for 5 *minutes*.

As mentioned previously, we observed the formation of an elastic network from filaments under the stated experimental conditions (Fig 4). The easiest way of characterizing their motion is by approximating their bending with a harmonic potential [45]. Using a nonlinear Gaussian fit (in the form of: *A* *exp*(–(*x*–*x*_0_)^2^/(2*σ* ^2^))) of the tracer position distribution in the direction perpendicular to the bundle axis (y-direction), one can extract the spring constant as *k*_*y*_ = *k*_*B*_ *T*/*σ* ^2^, where *k*_*B*_ is the Boltzmann constant and *T* is the temperature of the experiments (*T* ≈ 22°*C*). While this analysis is very simplistic, it eliminates the high numerical error in calculating the creep compliance, and the even higher errors associated with estimating the complex shear modulus [37].

**Fig 5:**
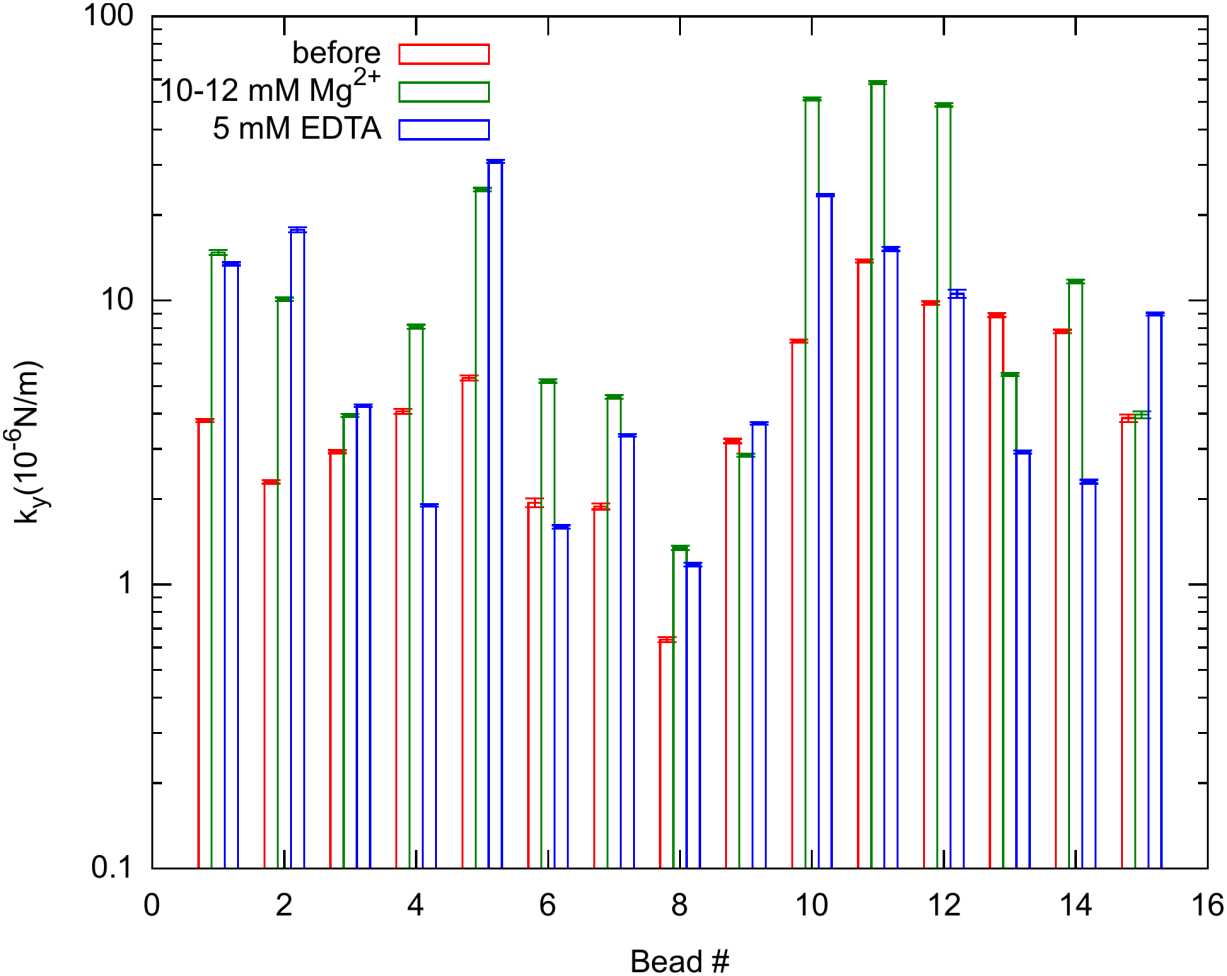
Apparent spring constants. *k*_*y*_ perpendicular to the bundling axis, before bundling (red), after bundled by 10–12 *mM* *MgCl*_2_ ions (green) and 3 *hours* after rinsing with the ion removal buffer (blue). There are three clear cases when the stiffness of the bundle returned back or became less than the original value. In all other cases, the bundles remained stiffer than the initial value. (Semilogarithmic plot of 15 bead positions measured in 3 independent experiments. The errors are estimated from the Gaussian fit of the position distributions.)

Fig 5 compares the *k*_*y*_ values obtained using nonlinear fitting before bundling, after bundling and 3 *hours* after EDTA treatment. After washing the loosely bound *Mg* ^2+^ ions away, the elasticity remains close to the values obtained after bundling in general, much higher than their initial values indicating that the bundling process was not reversed. In four cases however, *k*_*y*_ returned or was even less than its value before the divalent cations were introduced (e.g. bead numbers 1, 12, 14, 15).

It is important to note that this experiment has various sources of potential errors. Firstly, the actual concentration of actin in the buffer is unknown, thus the quantity of actin attached to the bead cannot be measured in this system. Subsequently, we cannot distinguish the extent of changes caused by filaments directly bound to the beads. However, fluorescence images taken at the three stages of the experiment, i.e. before adding magnesium, after bundling and after treating with the chelating agents, indicate a similar overall trend in the network in agreement of our previous observations (see S1–S3 Figs) [22]. Furthermore, there are several events, which may result in breaking of filaments, including photobleaching or mechanical vibrations, which would act to decrease the apparent spring constant.

Another potential source of error is the bundling of filaments due to oxidation. Our buffers were produced with degassed water, but no further precautions were taken to prevent oxygen from diffusing into the channels. The oxidative effect can manifest by initiating disulfide formation between actin monomers, which has been reported to affect the polymerization after exposing G-actin to air for several days [46]. As our experimental time course is only on the order of hours, and we maintained constant flow throughout the pumping time, which should eliminate the accumulation of radicals in the buffer, we do not believe oxidation plays a role in our system.

### Crosslinking Dynamics

The experimental results above provide supporting evidence that actin bundles are strongly interacting, possibly entrapping the cations between the filaments. Such a mechanism has also been suggested based on X-ray diffraction studies of actin [47]. In 3-dimensional gels, the divalent ions are mixed into the polymerization buffer, resulting in any time-dependent bundling to be obscured by the time dependence of actin polymerization. Using only low ion concentrations (a few *mM*) may result in the depletion of the ion stock around the filaments, thereby making the effect even less observable. However, our 2-dimensional system with constant flow supporting a constant background concentration should allow for the detection of time-dependent bundling.

We have chosen *Mg* ^2+^ concentrations from 2–12 *mM* the critical value we observed in our previous qualitative experiments [22]. The bundling process was again followed using bound PS particles and measuring their thermal motion, characterized by the apparent spring constant in the perpendicular direction to the bundle axis (*k*_*y*_).

**Fig 6:**
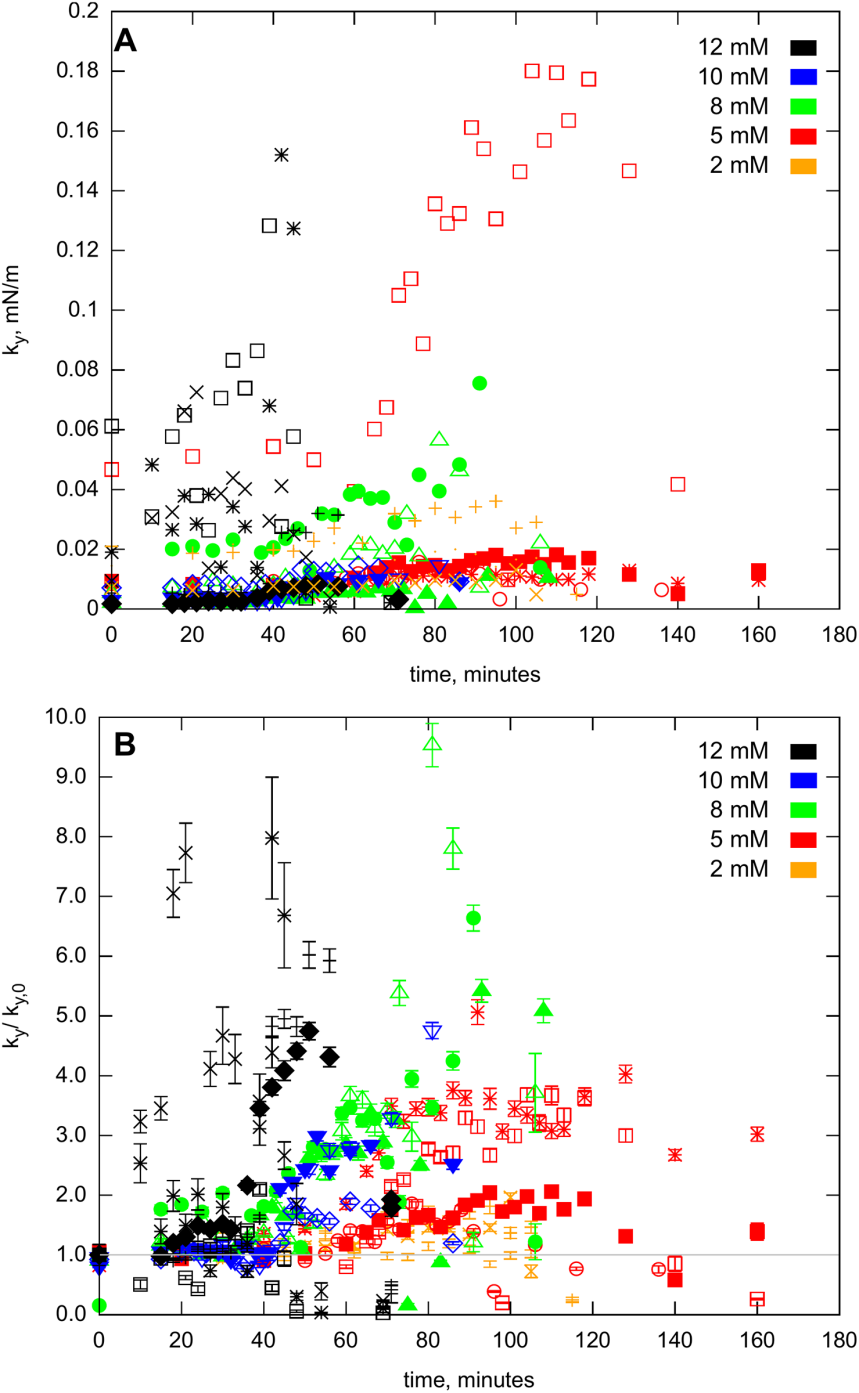
Temporal progression of the elastic force constant. *k*_*y*_ at *Mg* ^2+^ concentrations between 2–12 *mM*. (A) the absolute values (errorbars smaller than the symbols; not shown), and (B) normalized to the average of the first 3 points (at time 0).

The time-dependent variation of the apparent spring constant *k*_*y*_ is presented in Fig 6. A transition is clearly visible as the actin filaments form bundles; however, the individual datasets do not clearly group according to the magnesium ion concentration. The varying amount of actin filaments in the individual bundles and the number of filaments attached initially to the tracer beads results in a broad distribution of data at the outset of the experiments (Fig 6A). To decrease this effect, we normalized each dataset to the average of the first three measurement points, where the bundling was not yet present. The data could then be grouped together, yet still displaying the general concentration dependence.

The normalized data (Fig 6B) shows that the lowest magnesium concentrations of 2–5 *mM* resulted in the slowest bundling rate, while the highest, 12 *mM*, resulted in the fastest. However, the distribution of the data still does not allow for distinguishing a clear trend between the individual ion concentrations. Again, this broad distribution may be the result of various events, such as breaking off or adding new filaments to the bundle during the process. Occasionally, the zipping process, possibly combined with photobleaching, breaks enough of the bundle causing a sudden drop off in the data trend.

An important difference in our experiments vs. classic actin-gel experiments is that the continuous flow in the microfluidic chamber maintains the background concetration at a constant level. A previous study on the flow in this type of chamber determined that the flow speed is on the order of 40 *μm*/*s* at the pillar tops [39], which is just enough to supply fresh ions to the reaction, but locally, at the length scale of the filaments, diffusion still dominates. Additionally, because of the higher flow speed (about 50 *μm*/*s*) in the open volume between the pillar field and glass coverslip, a slight concentration gradient may occur. The difference, however, is not high enough to assume a significant concentration distribution within the channel. Lastly, while the data trends do not fall into well-defined groups according to the magnesium concentration, these data still support our hypothesis that the magnesium ions cause bundling below the previously estimated critical concentrations in a time-dependent manner.

## Conclusion

In summary, we have investigated the bundling behavior of actin networks at low concentration of divalent cations, specifically magnesium ions. In order to resolve the apparently contradicting results in the literature and within our own experiments, we hypothesized that the electrostatic interactions lead to the binding of magnesium to actin, whereby the ions may become entrapped between the filaments, resulting in an irreversible process. If such a process exists, the bundling kinetics should be time-dependent, especially at low ion concentrations, and the bundles should remain stable after removing magnesium from the buffer. To assess the bundling kinetics, we performed experiments with partially-oriented actin networks anchored on the tops of micropillars. Using *Mg* ^2+^ ions, we observed elastic characteristics of the filament bundles, with a broadened elastic plateau in comparison to data published in the literature for 3-dimensional actin gels. This specific response observed here is due to our experimental configuration for two reasons. First, binding the filaments to the pillars hinders diffusion, thereby eliminating the long-term break down of the plateau. Secondly, because the test particles were bound to the filaments, the motion of actin was observed rather than the diffusion within the filament network. Removing the divalent cations from such systems showed no reversibility of the bundling, indicating the possible entrapment of ions between the filaments. In comparison to the very weak (sub piconewton) forces driving actin bundling [25], this kinetic stability suggests a strong hysteresis in the bundling process.

Investigating the time dependence of bundling, we found a clear influence of *MgCl*_2_ on the global structure of the bundles at low concentrations (2–12 *mM*) of *MgCl*_2_, resulting in changes to the elasticity of the actin network. These results support our hypothesis that actin filaments do not behave as simple linear polyelectrolytes. Instead, ion-initiated actin bundling seems to occur along a very shallow interaction potential, but results in the entrapment of the ions, which stabilizes the formed bundle. The cumulative concentration of ions is determined by the background concentration, the local speed of flow or the diffusion of the ions, and the time it takes for the ions to first reach the filaments. It is important to keep in mind that the measured data could be influenced by several confounding factors, including the possibility that the beads themselves contribute to the bundling, increasing the apparent spring constant of the local network. However, fluorescence images indicate a global effect, even in the absence of test particles. Thus, we cannot conclude quantitative kinetic information from this experimental set-up, but we can confirm the existence of the phenomenon.

## Supporting Information

S1 Fig Fluorescence images of actin networks. General view of an actin field before crosslinking. The images are averaged time recordings of c.a. 15 frames to provide better contrast. (Pillar diameters are 5 *μm*.)
(PNG)

S2 Fig Fluorescence images of actin networks. Same as in S1 Fig, after crosslinking with 12 *mM* of magnesium ions.
(PNG)

S3 Fig Fluorescence images of actin networks. Same as in S1 Fig, 3 *hours* after removing the ions.
(PNG)

S1 Data A zip archive, containing all particle trajectory data used in this article, including configuration and plot files used for processing. See the Readme.txt file within.
(ZIP)
