# Supplementary material for: Reversibility and Viscoelastic Properties of Micropillar Supported and Oriented Magnesium Bundled F-Actin
Source: PLoS One. 2015 Aug 31;10(8):e0136432. doi: 10.1371/journal.pone.0136432 (PMC4556452; doi:10.1371/journal.pone.0136432)
Supplement: S1 Data — See the Readme.txt file within. (ZIP) [file pone.0136432.s004.zip › Rheology-raw-data/Long-time-results/J0-inv-fitted.pdf]

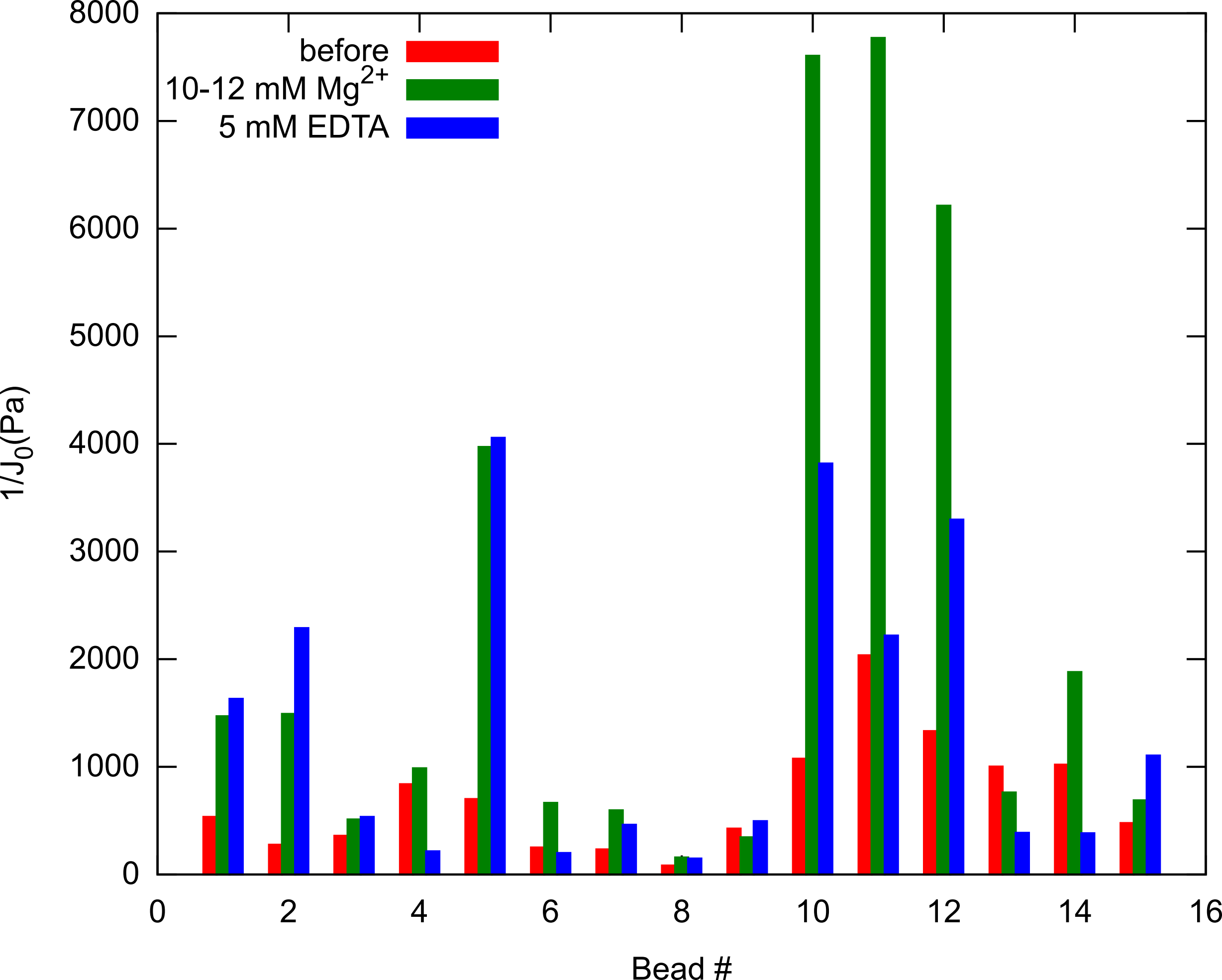

before
10-12 mM Mg2+
5 mM EDTA
1/J0(Pa)
Bead #
0
1000
2000
3000
4000
5000
6000
7000
8000
0
2
4
6
8
10
12
14
16
