# Supplementary material for: Reversibility and Viscoelastic Properties of Micropillar Supported and Oriented Magnesium Bundled F-Actin
Source: PLoS One. 2015 Aug 31;10(8):e0136432. doi: 10.1371/journal.pone.0136432 (PMC4556452; doi:10.1371/journal.pone.0136432)
Supplement: S1 Data — See the Readme.txt file within. (ZIP) [file pone.0136432.s004.zip › Rheology-raw-data/Long-time-results/Jfitted.pdf]

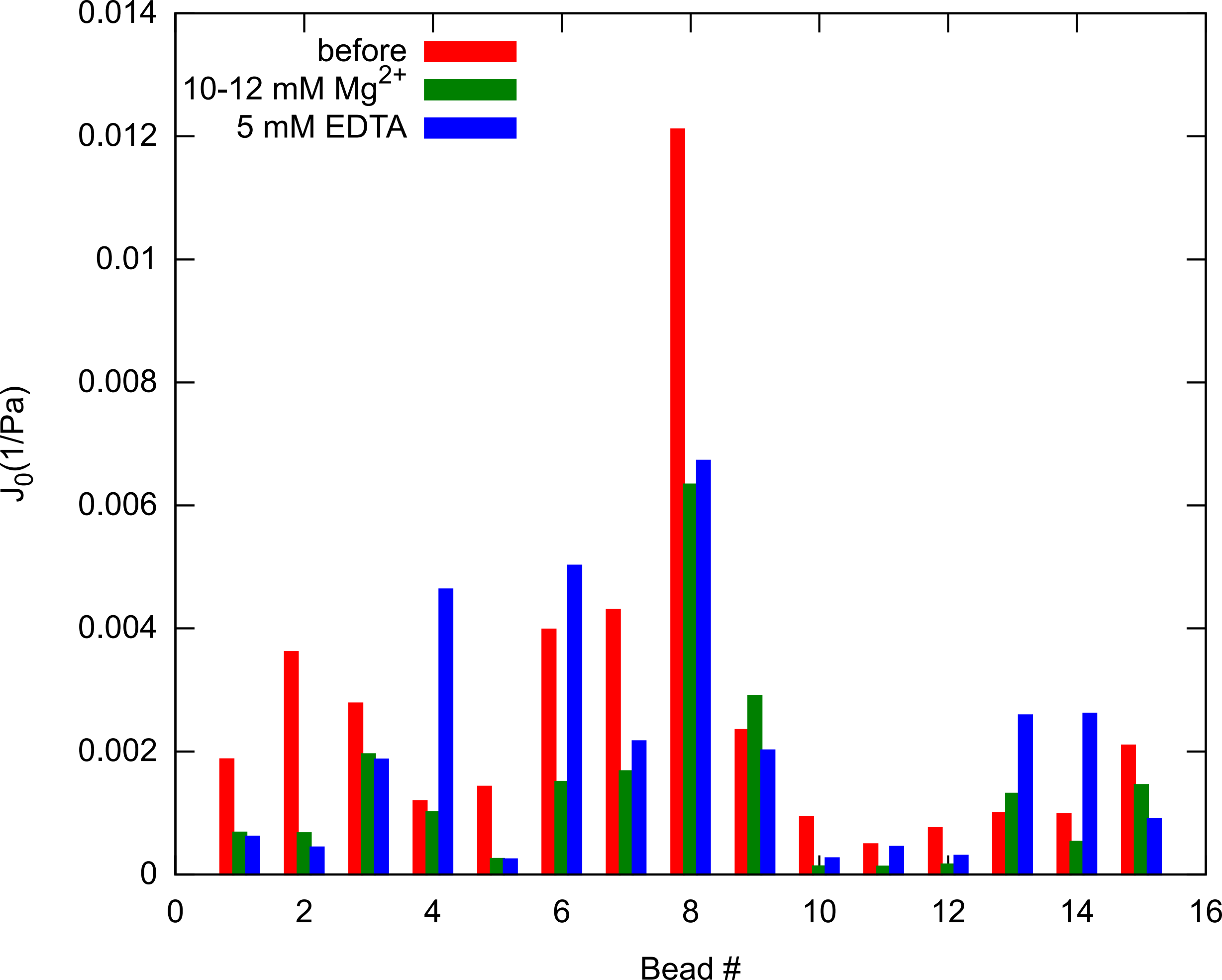

before
10-12 mM Mg2+
5 mM EDTA
J0(1/Pa)
0.014
0.012
0.01
0.008
0.006
0.004
0.002
0
0
2
4
6
8
10
12
14
16
Bead #
