# Supplementary material for: Reversibility and Viscoelastic Properties of Micropillar Supported and Oriented Magnesium Bundled F-Actin
Source: PLoS One. 2015 Aug 31;10(8):e0136432. doi: 10.1371/journal.pone.0136432 (PMC4556452; doi:10.1371/journal.pone.0136432)
Supplement: S1 Data — See the Readme.txt file within. (ZIP) [file pone.0136432.s004.zip › Rheology-raw-data/Long-time-results/ky-log.pdf]

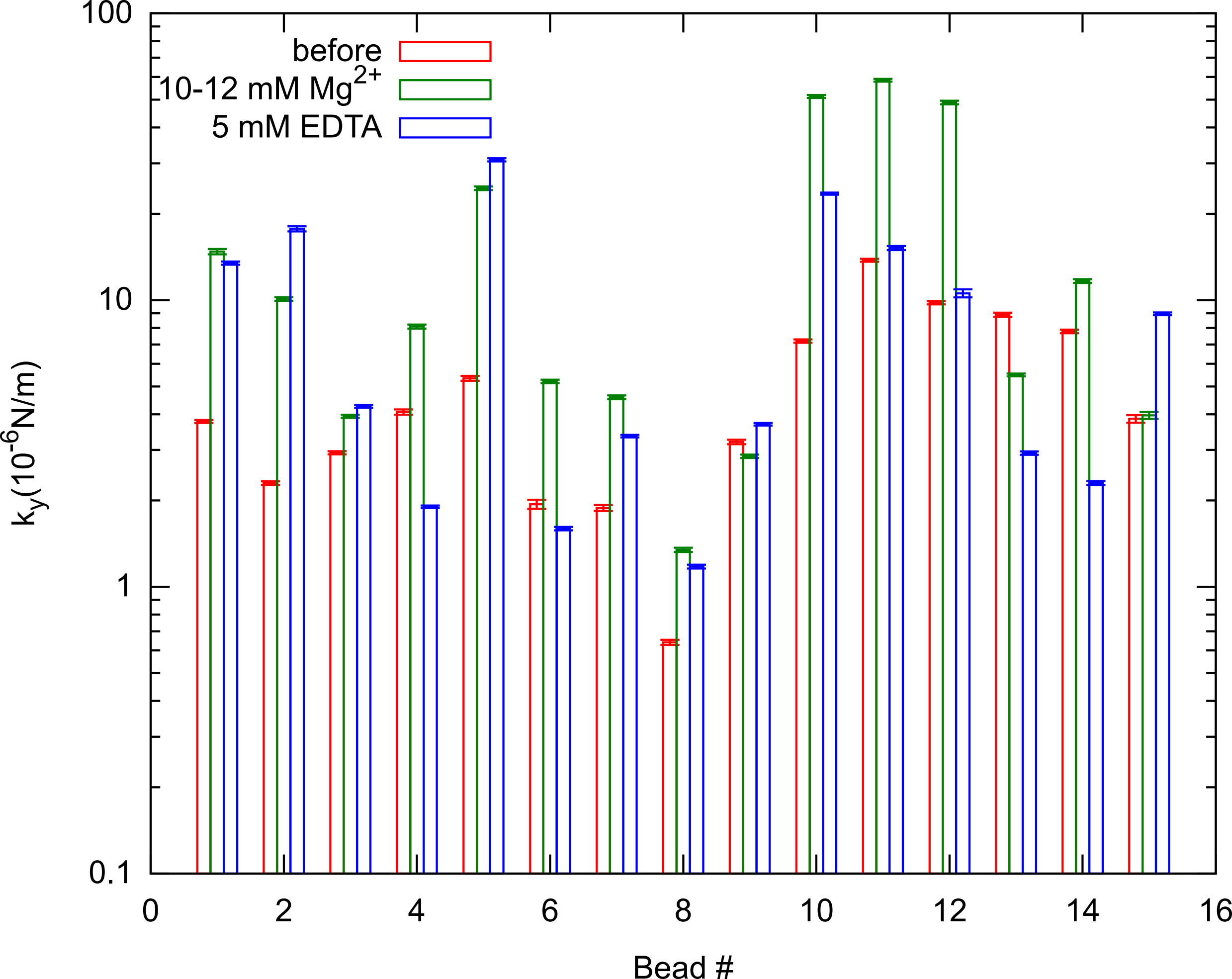

before
10-12 mM Mg^{2+}
5 mM EDTA
100
10
1
0.1
$k_y(10^{-6}N/m)$
0
2
4
6
8
10
12
14
16
Bead #
